# Effect of insulin therapy and dietary adjustments on safety and performance during simulated soccer tests in people with type 1 diabetes: study protocol for a randomized controlled trial

**DOI:** 10.1186/s13063-017-2078-1

**Published:** 2017-07-20

**Authors:** Javier Calvo-Marín, Gabriel Torrealba-Acosta, Matthew Campbell, Jesse Gaboury, Ajmol Ali, Chih Hao Chen-Ku

**Affiliations:** 1 Division of Endocrinology, Hospital San Vicente de Paul, Heredia, Costa Rica; 2 Neuromodulation Center, Spaulding Rehabilitation Hospital, Harvard Medical School, Boston, MA USA; 3 School of Sport, Leeds Beckett University, Leeds, West Yorkshire UK; 4 School of Sport and Exercise, College of Health, Massey University, Albany, New Zealand; 5 Department of Pharmacology and Clinic Toxicology, University of Costa Rica – Division of Endocrinology, Hospital San Juan de Dios, San José, Costa Rica

**Keywords:** Type 1 diabetes, Exercise, Hypoglycemia, Insulin, Continuous glucose monitoring

## Abstract

**Background:**

Despite the reduction in glycemic derangement in patients with type 1 diabetes mellitus (T1D) through dietary and therapeutic adjustments implemented before, during and after continuous exercise, evidence for its effectiveness with intermittent forms of exercise, such as soccer, is still lacking.

**Methods/design:**

We designed a study protocol for a randomized, crossover, double-blinded, controlled trial, for the evaluation of the effect that a strategy of dietary and therapeutic modifications may have on safety and performance of persons with T1D in soccer training sessions and cognitive testing. Inclusion criteria comprise: age older than 18 years, more than 2 years since T1D diagnosis, low C-peptide level, a stable insulin regimen, HbA1c less than 9.0% and regular participation in soccer activities. Our primary outcome evaluates safety regarding hypoglycemia events in patients using dietary and therapeutic adjustments, compared with the performance under the implementation of current American Diabetes Association (ADA) usual recommendations for nutritional and pharmacological adjustments for exercise. Additionally, we will evaluate as secondary outcomes: soccer performance, indexed by performance in well-established soccer skill tests, cognitive functions (indexed by Stroop, digital vigilance test (DVT), Corsi block-tapping task (CBP), and rapid visual information processing (RVIP) tests), and glycemic control measured with a continuous glucose monitor (CGM).

**Discussion:**

Dietary and insulin adjustments standardized under a 4-step method strategy have never been tested in a clinical trial setting with intermittent forms of exercise, such as soccer. We hypothesize that through this strategy we will observe better performance by persons with T1D in soccer and cognitive evaluations, and more stable control of glycemic parameters before, during and after exercise execution, indexed by CGM measurements.

**Trial registration:**

ISRCTN, ISRCTN17447843. Registered on 5 January 2017.

**Electronic supplementary material:**

The online version of this article (doi:10.1186/s13063-017-2078-1) contains supplementary material, which is available to authorized users.

## Background

The importance of encouraging people with type 1 diabetes mellitus (T1D) to be physically active is well-established when pursuing goals such as overcoming a sedentary lifestyle, improving weight control, ameliorating pre-existing cardiovascular disease, and improving quality of life [1]. The American Diabetes Association (ADA) recommends that individuals diagnosed with T1D participate in regular exercise in attempts to improve different health outcomes such as weight loss and optimal glycemic control. However, routine practice of this intervention is significantly hampered by the risk of exercise-induced hypoglycemia [2]. Additionally, it is frequently observed that people with T1D, concerned about having hypoglycemic events while exercising, tend to increase their caloric intake excessively, eventually leading to vexing glucose variability and further challenging the execution of physical activity [3].

Prior evidence has focused on the effects of certain therapeutic and dietary adjustments on patients’ performance when they participate in sports that involve continuous physical exercise [4]. However, recent publications demonstrate safety, evidenced by protection in terms of rates of hypoglycemic events by reducing degludec insulin dosage in subjects with T1D, even with different exercise intensity and modality (indexed by lactate threshold and potentiometers) [5].

Therefore, it is pertinent to study similar pharmacological and dietary interventions but with intermittent exercise. This exercise modality is more demanding on the body than continuous physical activity of the same average intensity [6], with a greater rise in body temperature (0.3 °C) and an added elevated blood lactate concentration when compared at a given percentage of VO_2_ max [7]. Concomitantly, sweat loss during intermittent exercise can exceed sweat loss during continuous exercise [8], thereby increasing the reliance on carbohydrate (CHO) as a substrate during this type of physical activity [9].

In contrast to continuous exercise that only comprises the workload intensity and the total duration, intermittent exercise consists of five main components: peak work load intensity, peak workload duration, recovery load, recovery duration, and the mean load [4]. A clinical trial comparing continuous with intermittent physical activity in subjects with T1D showed that a lower amount of exogenous carbohydrate is required to maintain euglycemia during intermittent compared with continuous activity, and this finding was not related to increased hepatic glucose output but to significantly lower glucose disposal in intermittent compared with continuous activity. Metabolically, this was paralleled by increased levels of counter-regulatory hormones (mainly growth hormone and catecholamines), and substantially higher levels of lactate in intermittent activity [10].

A realistic recommendation is that exercisers with diabetes mellitus should take in adequate carbohydrate, along with sufficient (albeit likely reduced) insulin before, during, and after prolonged moderate-intensity or high-intensity workouts to maintain and restore muscle and liver glycogen and blood glucose, especially during that window of opportunity for glycogen repletion right after the end of exercise (within 30 min to 2 h after) [11].

It is also important to note that the speed with which insulin is absorbed and its onset of action may be increased by both local heating and massage at or near the application site. In addition to changes in insulin dosage and CHO intake, exercise itself may be used to prevent hypoglycemia. For instance, performing a brief (10 s) maximal intensity sprint either before or after a moderate intensity exercise session may protect against hypoglycemia onset, at least in the short run [11].

The effect that the modifications on blood glucose levels before, during, and after exercise have on the performance of athletes in this type of sport has not been accurately established, and evidence addressing this hypothesis is scarce [1]. To our knowledge there is only one study [8] in adolescents with T1D that found that hypoglycemia, but not hyperglycemia, impaired both the patients’ sports performance (tennis, basketball, or soccer skills) and their cognitive function. However, this trial had some important limitations including the fact that the sport performance tests were of a relatively short duration and did not demonstrate how blood glucose levels affected mental and physical stamina [12].

Campbell et al. [2] developed a 4-step method that succeeded in protecting participants with T1D from both hypoglycemia and hyperglycemia throughout exercise periods and the 24-h interval following exercise sessions [2]. This method includes the following interventions: (1) 75% reduction of pre-exercise rapid-acting insulin dose (or 50% if intermittent exercise) given before a meal containing 1 g carbohydrate · kg^-1^ 60 min prior to physical activity [13]; (2) post-exercise meal containing 1 g low-glycemic index (GI) carbohydrate · kg^-1^ 60 min after physical activity with 50% reduction on rapid-acting insulin dose [14]; (3) consumption of a bedtime snack containing 0.3 g carbohydrate · kg^-1^ also with a low GI and omitting prandial insulin administration for this meal [15]; and (4) 20% reduction of basal insulin across the day when physical activity is performed [2].

In clinical trials, this 4-step method has demonstrated that it is possible to optimize blood glucose level during and after exercise by making mealtime adjustments to both rapid-acting and basal insulin, and to pre-exercise and post-exercise carbohydrate composition [2]. However, previous clinical trials have focused on evaluating patients’ performance in continuous forms of sports like running or cycling, whereas evidence supporting this 4-step strategy in intermittent activities such as soccer, tennis, and basketball, is still lacking.

Therefore, we designed a protocol for a randomized, crossover, double-blinded, controlled trial to evaluate the effect that this 4-step strategy for blood glucose control will have on patients’ performance in a series of soccer training sessions and cognitive assessments, compared with usual care recommendations. Moreover, in this study protocol, we introduce the evaluation of this 4-step method in a more realistic “real world” scenario of sports practice, with simulation exercises based on the soccer gameplay field. We hypothesize that participants using the 4-step method will yield a better performance both in soccer-simulated sessions and in cognitive function evaluations when compared to the implementation of the usual ADA recommendations. We also expect fewer glycemic derangements and better glycemic control before, during, and after the performance of physical activities, when applying the 4-step method on the participants.

## Methods

### Research aim

The aim of this research is to assess the safety (evaluated as rate of hypoglycemia) that this 4-step strategy has on patients with T1D after completing a series of simulated soccer tests. As exploratory analysis, we will compare the performance of the skills required to achieve a good score in the selected test. We will also aim to identify an optimal pre-exercise blood glucose level associated with a better performance in simulated soccer tests. Finally, we will evaluate the effect of this 4-step strategy for blood glucose level control in the cognitive performance (indexed by neuropsychological evaluations testing executive functions, attention, working memory, psychomotor speed, and visual-spatial short-term memory) in patients diagnosed with T1D.

### Trial design

This is a randomized, crossover, double-blinded (subject and evaluator), controlled trial. Participants will be randomized to the order of the interventions assigned (real 4-step method versus usual recommendations (sham 4-step method)), leaving a washout period of 3 weeks for repeating assessments after switching groups. We follow the standard protocol items: recommendation for interventional trials (SPIRIT) guidelines to cover all sections involved in this protocol (see Additional file 1).

### Participants

Eligibility criteria for this trial will be focused on selecting patients with T1D that have a stable insulin regimen and glucose control and are engaged regularly in exercise and physical activities, as this is the target population to whom we would like to generalize the results obtained. Table 1 lists the inclusion and exclusion criteria for this trial. Participants are required to be able to understand instructions for motor and cognitive evaluations and to provide informed consent, in order to be enrolled.

**Table 1:** Inclusion and exclusion criteria

Inclusion criteria	Exclusion criteria
Participants older than 18 years More than 2 years of established T1D Stable insulin regimen in the past 6 months indexed by less than 20% change in total insulin daily dose HbA1c less than 9.0% Weekly physical activity of 90 min or more Participation in recreational or competitive soccer-related activities, at least four times per month Participants able to provide informed consent	Any treatment regimen different from continuous subcutaneous insulin infusion or multiple daily insulin injections Any use of medications (other than insulin) known to affect glycemic control including oral or parenteral steroids (except for inhaled steroids), metformin, SGLT2 inhibitors, GLP.1 agonists, thiazolidinedione, sulfonylureas, DPP-4 inhibitors, or any other oral antidiabetic therapy Detectable C-peptide in serum Cognitive impairment comprising any substantial decrease in alertness, language reception, or attention that might interfere with understanding instructions for motor and cognitive testing, not including hypoglycemia unawareness Chronic kidney disease (glomerular filtration rate less than 90 mL · min^-1^) Chronic liver failure (Child-Pugh B or C) Advanced diabetic retinopathy Pregnancy Color blindness

*T1D* type 1 diabetes, *SGLT-2* sodium glucose cotransporter 2, *GLP1* glucagon like peptide 1, *DPP-4* dipeptidyl peptidase 4

### Recruitment

Recruitment of participants will be based mainly on referral by endocrinologists, internal medicine physicians or other professionals involved in the care of patients with T1D. However, we are also seeking to recruit patients in collaboration with members of the Diabetes and Endocrinology Medical Association in Costa Rica (ANPEDEM) and through the monthly meetings of the People with Diabetes National Foundation in Costa Rica (Dia Vida Foundation) and social media advertisements. Additionally, Institutional Review Board (IRB)-approved flyers with the trial information will be situated near the lobby of clinics and hospitals where patients with T1D attend for monitoring.

The trial research assistant will contact candidates through personal or telephone interview and will complete a pre-screening evaluation (see Table 3) form assessing all of the inclusion and exclusion criteria. Participants that fulfill the eligibility criteria will be defined as potential participants of the clinical trial, and will receive the informed consent form by mail or email. Afterwards, a face-to-face meeting will be scheduled and during this session (visit 1), the principal investigator will explain the methodology and the design of the study in detail. The subject will also be asked to give their written informed consent during this visit, after they have asked questions and/or expressed their concerns.

### Baseline assessments

Baseline assessments will be carried out by a physician and will take place after consent has been obtained and before randomization. During this visit (visit 1), a physician will complete an evaluation with clinical data about the subject’s physical activity routine, chronic diabetes mellitus control, history of hypoglycemia, and body mass index (BMI) measurement. Additionally, the following laboratory evaluations will be performed: fasting plasma glucose, serum C-peptide level, HbA1c and urinary albumin excretion in a spot urine sample. Following all baseline assessments the subject will be randomized to their respective arm.

One week after randomization, a second visit (visit 2) will take place during which a certified physical education teacher and a soccer trainer will give the participants a theoretical and practical explanation of the training session exercises that will be conducted in the trial. During this visit, the participants will execute a short version of the simulated soccer exercises to become familiar with them. Additionally, participants will receive an educational session with an endocrinologist with all of the indications to complete the blood glucose management through insulin and dietary adjustments.

### Intervention

The intervention will consist of either following the recently published ADA usual recommendations of care [12–16] before, during and after exercise (sham 4-step method), or the real 4-step method (a meal 60 min pre-exercise, with 50% of insulin dose; a low GI meal 60 min post-exercise, with 50% of insulin dose; a low GI snack at bedtime before sleep, omitting the insulin dose; and 20% reduction in the basal insulin dose). On the day after exercise, participants will return to their usual full basal insulin dose. Moreover, the type of insulin used in the clinical trial will not differ from that used by the participants prior to enrolling in the study. The ADA guidelines suggest that short-acting insulin is reduced depending on the mean exercise intensity in both exercise modes [16].

There will be specific evaluation of the intermittent modality of exercise, given that the main objective that guides this exercise protocol is not to demonstrate differences in the response of subjects with T1D to diverse exercise modalities, such as continuous versus intermittent exercise. On the contrary, the intervention will evaluate the impact in terms of the safety and performance of a protocol that sets forth insulin dosage along with carbohydrate intake diet modifications, compared with usual-care recommendations stated by guidelines for the standard of care. It is worth adding that other trials have evaluated the response of subjects to the 4-step method [2, 14, 15] in protocols that had evaluated continuous physical activity.

#### Active intervention: real 4-step method

Participants will come to the laboratory at 7:00 a.m. and we will first obtain a resting, fasted venous blood sample (via venipuncture) for the laboratory evaluations previously listed. The iPro2 Professional continuous glucose monitor (CGM) with Enlite sensor (Medtronic, MN, USA) will be installed at that time. Participants will then consume a standardized cereal-based breakfast meal (sugar-coated corn flakes, peaches, and semi-skimmed milk; 1.3 g carbohydrate · kg^-1^ body mass). At 1:00 p.m. participants will consume a standardized pasta-based lunch meal (pasta, tomato-based sauce, cheddar cheese, olive oil; 1.3 g carbohydrate · kg^-1^ body mass). Between the morning and evening visits, participants will record their self-measured glucose control every 2 h.

At 5:00 p.m. participants will arrive for the evening training session. Before starting this session, they will self-administer a 50% reduced pre-meal rapid-acting insulin dose into the abdomen (the injection site will be at the mid-point between the iliac crest and the navel). Immediately after receiving this insulin dose, participants will consume a pre-exercise carbohydrate bolus (sugar-coated corn flakes, peaches and semi-skimmed milk; 1.0 g carbohydrate · kg^-1^ body mass). Following this bolus, participants will remain rested for 60 min, at which point a finger stick sample for glucose testing will be drawn immediately before starting with the training sessions.

At 60 min post-exercise, participants will self-administer a pre-meal rapid-acting insulin dose reduced by 50%, into the abdominal site contralateral to the previously administered pre-exercise rapid-acting insulin injection. After receiving this insulin dose, participants will consume a carbohydrate-based meal (basmati rice, tomato-based sauce, turkey breast, and an isomaltulose orange flavored drink (10% solution); 1.0 g carbohydrate · kg^-1^ body mass) designed to elicit a low GI (GI = 37) response [2].

Following this meal the participants will be discharged and will continue recording interstitial glucose values with the CGM until the subsequent morning visits. At 180 min following the post-exercise meal (240 min post-exercise), participants will consume a low-GI bedtime snack (low fat yogurt with honey tap; GI = 38), equivalent to 0.4 g carbohydrate · kg^-1^ body mass, and will omit rapid-acting insulin. In order to improve adherence, participants will be contacted before the bedtime snack to ensure compliance, and they will be encouraged to replicate their sleeping patterns as much as possible prior to the training sessions.

The basal insulin dose after exercise will be reduced by 20% of the pre-trial basal dose. Nevertheless, the timing will be maintained throughout as per each participant’s individual regimen. Twenty-four hours afterwards, participants will return to their usual full basal insulin dose.

At 8:00 a.m. on the morning following each training assessment, participants will return to the laboratory. Here we will obtain another fasting blood sample through venipuncture and subsequently provide them with a standardized breakfast meal (sugar-coated corn flakes, peaches, semi skimmed milk; 1.0 g carbohydrate · kg^-1^ body mass). With this meal, they will self-administer their full unchanged rapid-acting insulin dose.

Following the consumption of breakfast, the participants will be discharged from the laboratory and instructed to maintain CGM for an additional 11 h (i.e., 24 h from exercise cessation). Also during this time, they will continue to monitor their diet using a food record diary and weighing their foods. We will provide the participants with a smartphone application for the recording of this information (MyFitnessPal App).

#### Control intervention: usual recommendations (sham 4-step method)

Recently, ADA published a position statement about physical activity and diabetes [16]. This statement explained how the variable glycemic responses observed in association with physical activity makes it very difficult to provide uniform recommendations for both the management of food intake and the insulin dosing regimen. Nonetheless, several instructions in this document are intended to help prevent hypoglycemia. To prevent low blood glucose level during prolonged (>30 min), predominantly aerobic exercise, additional carbohydrate intake and/or reductions in the insulin dose are typically required [12–16]. In addition to the changes in insulin regimen and carbohydrate intake, a brief (10 s) maximal intensity sprint could be performed before or after the exercise session, as suggested in the ADA guidelines, to prevent exercise-induced hypoglycemia [12]. The position statement also recommends adjusting daily basal insulin dose with reduced prandial bolus insulin and low-GI carbohydrate feeding following evening exercise for those on multiple daily injections [12]. Finally, inclusion of a bedtime snack, overnight glucose checks, or alarms and automatic pump suspension may also be warranted [16].

Patients allocated to the control group, or sham 4-step Method, will receive current usual recommendations as stated by the ADA [16], but following the scheme established with the real 4-step method (see Table 2 for the comparison for both interventions). Prior to exercise, the CGM will be installed at 7:00 a.m., and they will receive their meals in the same amount and frequency as in the real 4-step method, consisting of a breakfast at 8:00 a.m., lunch at 1:00 p.m. and pre-exercise meal at 5:00 p.m. All of the three meals will have the same caloric content as in the real intervention, and there will be no insulin modification for the first two meals. Assuming that they will be performing moderately intense physical activity later in the day, for up to 60 minutes, this group will receive a rapid-acting insulin dose that will be 75% of their usual dose concomitantly with the 5:00 p.m. meal prior to the onset of physical activity.

**Table 2:** Scheme comparison of interventions delivered in the real and sham 4-step methods

Time		Real 4-step method	Control (sham) 4-step method
8:00 a.m.	Breakfast	Standardized cereal-based breakfast	Standardized cereal-based breakfast
	Insulin dose	Unchanged	Unchanged
1:00 p.m.	Lunch	Standardized pasta-based lunch	Standardized pasta-based lunch
	Insulin dose	Unchanged	Unchanged
5:00 p.m.	Pre-exercise meal	1 g · kg^-1^ CHO bolus	1 g · kg^-1^ CHO bolus
	Insulin dose	50% reduction	75% reduction
7:00 p.m.	Post-exercise meal	Low-GI 1 g · kg^-1^ CHO bolus	Medium-GI 1 g · kg^-1^ dinner
	Insulin dose	50% reduction	No reduction
10:00 p.m.	Snack	Low-GI 0.4 g · kg^-1^ CHO snack	Medium-GI 0.4 g · kg^-1^ CHO snack
	Insulin dose	No insulin administered	No insulin administered
Regular hour for basal insulin	Insulin dose	20% reduction	No reduction

*CHO* carbohydrates, *GI* glycemic index

One hour after finishing the exercise session, the participants will receive a carbohydrate-based meal with the same caloric content as the meal apportioned in the real interventional arm; however, we will not use low-GI food for this bolus. Contrarily we will give the participants traditional rice, tomato sauce, turkey breast, and water. With this meal we would be providing about 1.0 g of carbohydrate · kg^-1^ of body mass. We will not modify the insulin dosage administered with this meal, considering that the ADA guidelines do not suggest any specific change around this issue.

Participants will then go home where they will continue registering interstitial CGM. About 180 min after dinner (approximately 240 min after finishing the exercise session), the participants will be contacted to remind them to eat a snack before going to bed. However, this will differ from the real intervention in that this will not be a low-GI snack (200 mL of unsweetened orange juice and half a banana), and will be equivalent to 0.4 g of carbohydrate · kg^-1^ of body mass. Additionally, participants will not administer any insulin dose with this snack.

Current ADA guidelines [16] have not yet established any basal insulin dosage adjustment after intermittent exercise, and it remains for the treating physician to decide on a case-by-case basis. However, we have added a modification of 20% of the insulin dose in the interventional arm, based on results obtained from recent trials, an effect seen even with the newer basal insulin such as degludec. [5] Therefore, participants following the sham 4-step method will not receive the post-exercise modification in insulin dosage. However, in order to maintain blinding of the control group, we will provide the unchanged insulin dosage for them to administer in their own homes after the exercise session finishes, in preloaded and concealed insulin syringes, whereby they will not be able to ascertain the dose that has been served. Moreover, and with the aim of increasing adherence, the participants will be contacted through phone calls in order to remind them about eating the before-bedtime snack and to self-administer the insulin treatment provided by the trial.

At 8:00 a.m. on the morning following each training assessment, participants will return to the laboratory. Here we will obtain another fasting blood sample from them via venipuncture and subsequently provide them with a standardized breakfast meal (sugar-coated corn flakes, peaches, semi-skimmed milk). With this meal, they will self-administer their full unchanged rapid-acting insulin dose.

### Outcomes

A physician will follow the participants’ blood glucose levels un-blinded, a week before each assessment session. This control will be based on an email report of the daily blood glucose levels 7 days before the training session.

During each of the assessments (visits 3 and 6; see Table 3) a CGM will be installed at 7:00 a.m. and will be maintained until 48 h after the training session ends. During each of these visits the participants will perform the soccer performance evaluation at 5:00 p.m. These training sessions must be separated by 3 weeks, with the idea of reducing a possible “learning effect” associated with repeated workouts. In the 48 h following each training session, participants will be required to measure their blood glucose level eight times (i.e., 2 h after dinner, before going to bed, at 12:00 a.m., at 3:00 a.m., just before breakfast, 2 h after breakfast, just before lunch, 2 h after lunch, and just before dinner). They will also be provided with a form where they will be required to register the appearance and timing of symptoms of hypoglycemia. Blood glucose level will be evaluated using the Accu-Chek® Nano Glucometer (Roche Servicios SA, Costa Rica) 30 min before each assessment session, after each one of the workouts, and before both the basal and 24-h cognitive tests.

**Table 3:** Study visits summary table

Assessments and interventions	Pre-screening	Visit 1	Visit 2	Visits 3, 6	Visit 5	Visits 4, 7
Review of eligibility criteria	X					
Demographic data and clinical evaluation		X				
BMI measurement and laboratory evaluation		X				
Theoretical and practical explanation of training exercises			X			
4-Step method and usual care recommendations educational sessions			X		X	
Training sessions with simulated soccer exercises (LIST-P, LSST, LSPT)				X		
Cognitive evaluations				X		X
Efficacy of blinding assessment					X	X
Revision of self-monitoring of blood glucose after exercise session						X
Approximated time	30 min	60 min	90 min	120 min	90 min	60 min

*BMI* body mass index, *LIST-P* Loughborough Intermittent Shuttle Test Prescribed and Self-Paced, *LSST* Loughborough Soccer Shooting Test, *LSPT* Loughborough Soccer Passing Test

After completing the first evaluation, participants will change their interventional arm, and both groups will receive a second educational session (visit 5) from an endocrinologist, who will explain the details of their new dietary and pharmacological interventions. Also in this visit, we will assess the efficacy of blinding by asking participants in which interventional arm they think they are allocated, and how confident they are about their answer.

Furthermore, participants will be seen the day following each assessment visit (visits 4 and 7) during which cognitive testing and self-monitoring of blood glucose will be repeated. In the last visit (visit 7) we will assess the efficacy of blinding again by asking participants in which interventional arm they think they were allocated, and how confident they are about their answer.

All participants will be instructed to replicate and record their diets (MyFitnessPal App®) and maintain their current insulin regimen, without modifying the dose, injection site, or time of injection for 24 h before each experimental trial day (visits 3 and 6). Participants will also be instructed to maintain similar grades of physical activity during the trial, avoiding strenuous exercise during the previous 48 h and 48 h following the simulated soccer drills.

The assessment sessions will occur on two separate days (separated by 3 weeks) and consist of both cognitive and physical activity evaluations. During the physical activity assessments participants’ heart rate (HR) will be traced with a Garmin Vivofit 2 Bundle with HR monitor (Garmin^©^, Kansas, USA).

### Primary outcome

We will compare the percentage of participants with one or more confirmed hypoglycemic episodes (≤60 mg · dL^-1^) in the next 24 h following the assessment session. Other safety-related end points include changes in the mean and variability of 24-h plasma glucose based on CGM. Percentages of participants with hypoglycemic events will be calculated as categorized by the ADA^70^ and will be analyzed during the day (daytime; 6:00 a.m. to 11:59 p.m.), during the night (nocturnal; 12:00 midnight to 05:59 a.m.), and any time of day or night (24 h).

Precisely, hypoglycemic categories will include the following: (1) any hypoglycemia (events whether confirmed by self-measurement or not and whether symptomatic or asymptomatic); (2) documented symptomatic hypoglycemia (symptomatic events with plasma glucose ≤60 mg · dL^-1^); (3) asymptomatic hypoglycemia (events confirmed by plasma glucose ≤60 mg · dL^-1^ but without symptoms); and (4) severe hypoglycemia (events requiring assistance by another person to administer carbohydrate, glucagon, or other therapy). Confirmed or severe hypoglycemia will include documented symptomatic or asymptomatic events together with severe events.

### Secondary outcomes

The secondary outcomes will be comparisons of physical and technical performance in soccer training sessions between groups through the tests as subsequently described.

#### Loughborough Intermittent Shuttle Test – Prescribed and Self-Paced (LIST-P)

**Fig. 1:**
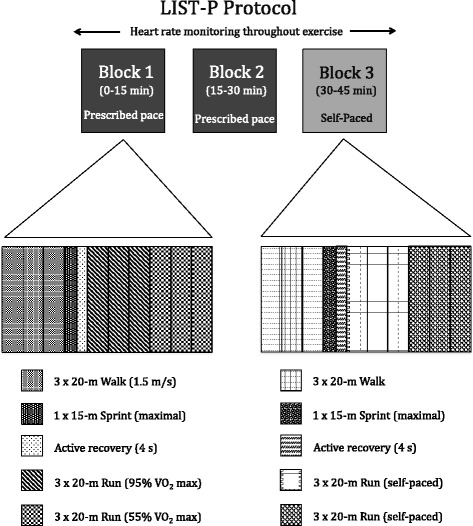
Schematic representation of the Loughborough Intermittent Shuttle Test - Prescribed and Self-paced (*LIST-P*). Modified from Ali et al. [17]

A modified and validated LIST protocol will be used as an equivalent to the intermittent physical activity that is performed during a soccer match. The LIST-P [17] requires participants to run back and forth between two lines 20 m apart. Each block of exercises consists of ~11 repeated cycles of walking, running (at a speed equivalent to 95% VO_2_ max), jogging (at a speed equivalent to 55%) and sprinting (defined as running to the maximum effort according to maximum VO_2_ max). Further, each cycle includes three repeated shuttles of walking, one 15-m sprint, three repeated shuttles of running and three repeated shuttles of jogging (Fig. 1). Audio signals will help to dictate the speeds at which participants will complete each shuttle.

The LIST-P will be modified to complete 45 min of the test. This reduction in test duration will be established in order to adjust the time of all evaluations to approximately 60 min. Consequently, this 1-h training session will have some similarity with previous clinical trials of continuous physical activity and T1D in terms of exercise duration, with the possibility of comparing the rate of hypoglycemic episodes and blood glucose control in participants. Therefore, the first two blocks of exercises (Blocks 1 and 2 in Fig. 1) will be performed at a prescribed-pace while the third and fourth blocks will be “self-paced” (Block 3 in Fig. 1).

Prescribed pace is established according to the VO_2_max reported during initial evaluation of the subject, when he or she was included in the clinical trial. On block 1 of LIST-P the prescribed pace includes repetitions of running at 95% VO_2_ max and 55% VO_2_ max (Fig. 1), whereas self-paced running will be defined and tailored specifically according to the subject’s preference. The auto-imposed pace will try to simulate conditions experienced during real gameplay, where subjects will run faster or slower depending on their physical capacity.

Prior to the beginning of the self-paced section, one of the investigators will ask participants to replicate the intensity and pattern of exercises done during the prescribed-pace period over the self-paced blocks of exercises. Evaluators will give no other verbal instructions during the self-paced blocks. The evaluators will record the duration of time spent on each activity mode (e.g., walking, running, jogging) for the self-paced cycle and for the total distance covered in the 15-min block.

Participants will consume 5 mL · kg^-1^ of a sugar-free sport drink (Powerade Zero ®, Coca-Cola Company) before starting exercise, and then 3 mL · kg^-1^ body mass of the same beverage every 15 min during the LIST-P protocol. Additionally, investigators will record body mass before and after each training session. Immediately after finishing each of the blocks from the LIST-P, participants will undergo psychometric testing using the perceived exertion scale (RPE) [18], the felt arousal scale (FAS) [19] and the feeling scale (FS) [20].

The participants will complete three 15-min blocks of the LIST-P punctuated by 4-min resting periods. After having completed the last block of LIST-P, participants will have 5 min of recovery time, and another finger-based capillary glucose sample will be obtained. After this the participants will complete the second round of Loughborough Soccer Passing and Shooting Tests (LSPT) as subsequently described.

#### Loughborough Soccer Passing Test

The LSPT has been validated previously in several populations [21] and it has been established to reflect the physical exercise and game dexterity required to execute soccer practice. For example, it evaluates and scores the player’s capacity for executing short passes correctly with their feet. Previous trials [21] that applied this test have been able to differentiate professional players from those still in training in minor leagues, thus evidencing the yield obtained from the test that further allows us to evaluate the subject’s performance in this protocol.

The LSPT requires the participants to start with the ball (Pioneer Pro #5, CICADEX, San José, Costa Rica) next to their feet, standing next to the central cone. One evaluator will start timing the test using a hand-held stopwatch (Casio Handheld Stopwatch Timer Model HS-3 V-1R, Casio Computer Co., Ltd, Tokyo, Japan), from the moment the ball is touched forward moving out of the inner rectangle.

A second evaluator will be responsible for verbally identifying the color of the next target (i.e., meaning the zone towards where the patient needs to kick the ball). The specific target will be verbally identified just before the participant completed the previous pass. This will be repeated until the subject kicks the ball towards the four established zones, a total of 16 times. The same examiner will be used for each role to eliminate inter-rater variability. The order of passes will be determined by one of four randomly generated trial orders, so that each trial will consist of eight long (green and blue) and eight short (red and black) passing combinations.

**Fig. 2:**
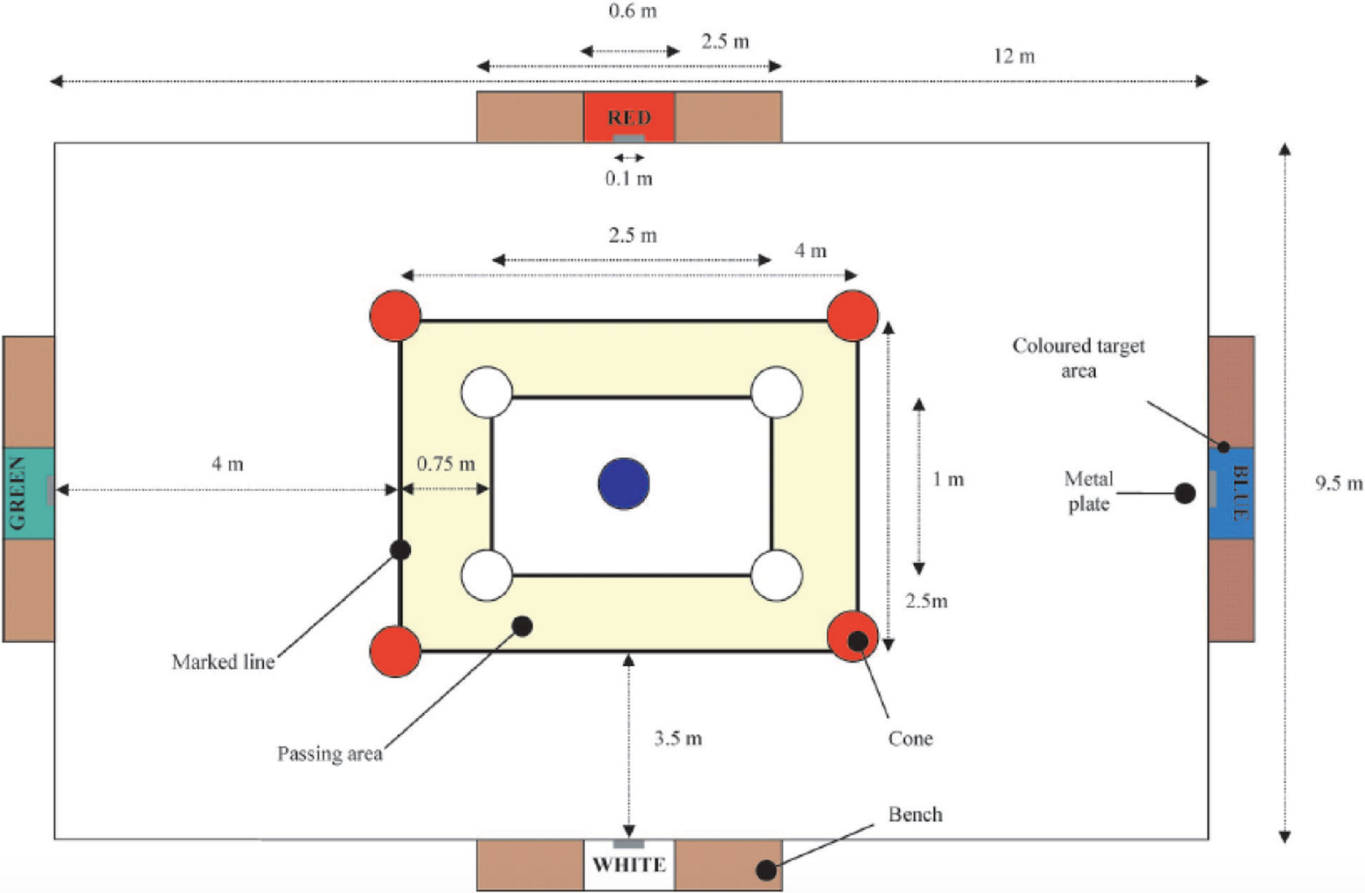
Schematic representation of the Loughborough Soccer Passing Test (LSPT). Ali A et al. [21]

The participants will be informed that the passes can only be executed from within the designated passing area, between the set of marked lines (see Fig. 2). They will also be instructed that upon retrieval from a previous pass, the ball must cross two of the inner marked lines before the next pass can be attempted. Furthermore, the players will be instructed that in order to attain the best performance in the LSPT they will have to execute the test as quickly as possible and make the fewest mistakes. The second evaluator will stop the clock when the last pass makes contact with the target area. The players will not know about the timing scores nor will they have any sort of performance feedback at any time during the data collection period.

The second examiner will also be responsible for recording the penalty time points accrued during the test. Thus, the examiner will stay in a position that all four target areas can be viewed. Penalty times will be appointed for the following errors:

1. 5 s for missing the bench completely or passing the ball towards the wrong bench
2. 3 s for missing the target area
3. 3 s for handling the ball
4. 2 s for passing the ball outside of the designated area
5. 2 s if the ball touches any of the cones
6. 1 s for every second spent over the designated 43 s needed to complete the test
7. 1 s will be subtracted from the total time if the ball hits the 10-cm strip in the middle of the target

Two trials of LSPT will be performed in each of the soccer training assessments, and the best score taken as the performance score.

#### Loughborough Soccer Shooting Test (LSST)

The LSST is also a validated test [21] that allows us to evaluate the capacity of a player (in this case our subjects) to score a goal within a simulated soccer gameplay scenario. This particular aspect of the test reproduces real-life conditions of gameplay, thus yielding a more real score of the subject’s performance.

**Fig. 3:**
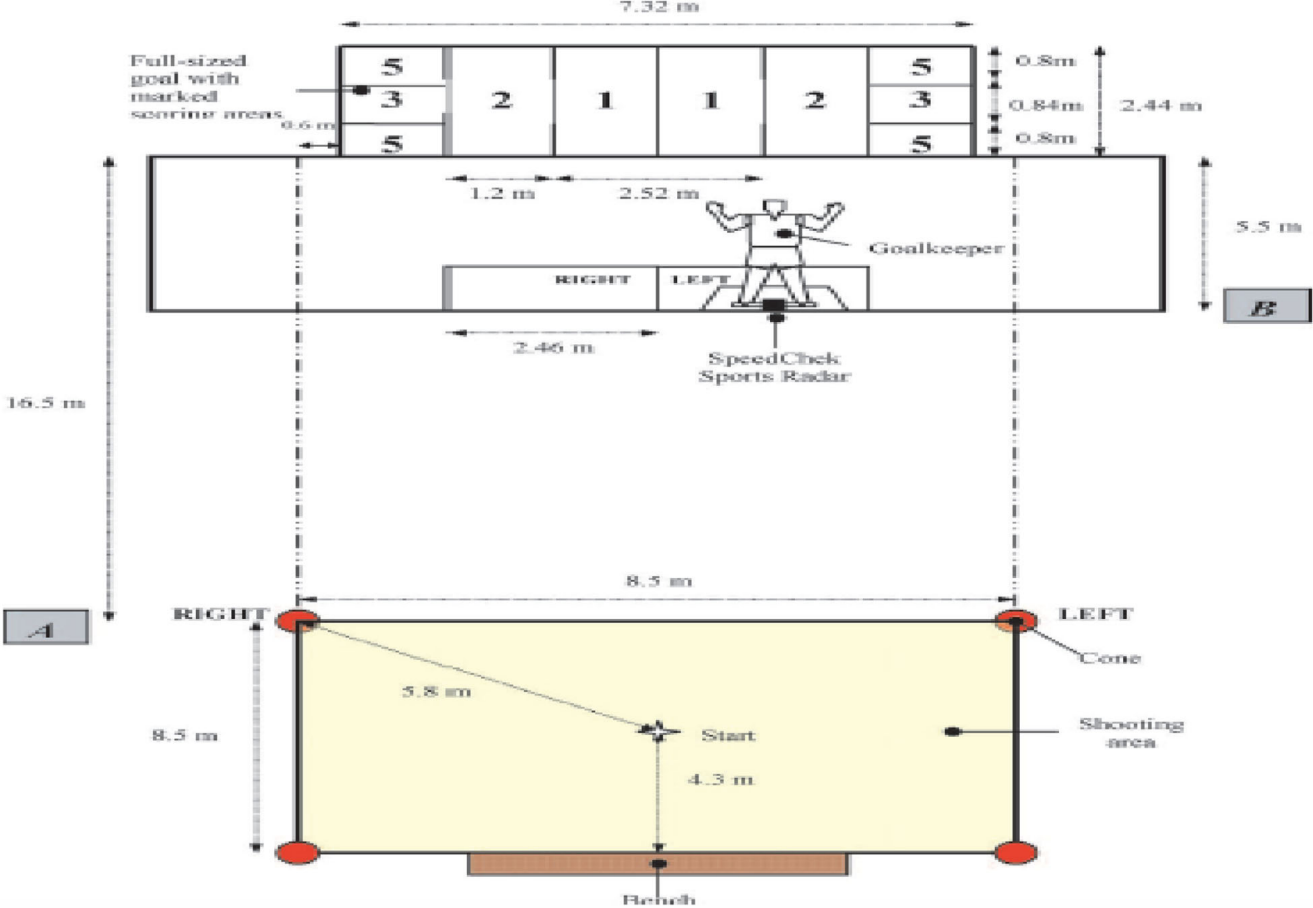
Schematic representation of the Loughborough Soccer Shooting Test (LSST). Ali A et al. [21]

Immediately following completion of the LSPT protocol, the participants will be evaluated with another capillary finger glucose level. After that, the trainer will proceed with the evaluation of the LSST (see Fig. 3).

For this test, the boundary lines will be measured and marked on the floor using a 5-cm yellow tape. The “shooting zone” will be a square area (8.5 × 8.5 m), with the nearest line marked at a distance of 16.5 m from the goal line. Four tall traffic cones will be placed on each corner of the shooting zone. A standard gymnasium bench will be placed in the middle of the far side of the zone to act as a rebound board. The full-sized goal, measuring 2.44 × 7.32 m, will be split into various scoring zones marked with a 5-cm tape (Fig. 3); this whole area will be drawn on a piece of polyethylene sheeting measuring 7 × 9 m.

To enhance the reality of the test, a static, life-size mannequin goalkeeper will be used. The figure will measure 1.9 m tall and 1.22 m at its widest point at the base and the arm span. The goalkeeper will be positioned 5.5 m in front of the goal line, and the side (right or left) will be changed depending on the experimenter’s instructions. The goalkeeper will be set on a base with four wheels to allow for ease of movement across the goal center during experimentation.

When completing the LSST protocol, the ball will be initially be placed on the marked cross in the middle of the shooting zone (Fig. 2). The starting position for the participant will be facing away from the goal within 0.5 m distance of the ball. On the evaluator’s call, the player will sprint to the appropriate cone, will touch the top of it, and then will return to play a “rebound” pass against the bench; the participant will turn and control the ball if necessary, and then will shoot at the goal while within the shooting area.

The player then will follow up his shot by sprinting past the goalkeeper. Each trial will consist of 10 shots with 1-min resting periods between each shot sequence. There will be six trial orders that will be randomly selected for each player. The goalkeeper mannequin will be positioned to the opposite side of the “left/right” call.

To prevent players from attempting shots at an “unrealistically slow” speed, a minimum value of 64 km · h^-1^ will be set for the shot to be valid for this test protocol. The Gamma SpeedTrac X radar gun (Gamma Sports, Pittsburgh, USA) equipment will be used to measure the shot speed.

During match play, the optimal placement of a ball to beat the opposing goalkeeper is at the top and bottom corners of the goal and the points associated with each scoring zone will reflect this (Fig. 2). Furthermore, a coach would normally encourage the players to shoot across the goalkeeper towards the open space of the goal [12]. To replicate this within the LSST, the players will be told that they can only score points if the ball strikes the open side of the goal, i.e., the half not covered by the mannequin goalkeeper.

Both left and right feet will be used during the LSST. The trial orders will be devised so that players have five shots for each foot per trial. The final score will be the mean of the total cumulative points accrued from all the shots on target. Those shots that were attempted from outside the shooting zone, took more than 8.5 seconds to complete, and/or were struck at less than 64 km · h^-1^, will be completely disregarded.

**Fig. 4:**
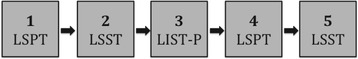
Schematic representation of the order of soccer assessments according to the protocol. *LIST-P* Loughborough Intermittent Shuttle Test Prescribed and Self-Paced, *LSST* Loughborough Soccer Shooting Test, *LSPT* Loughborough Soccer Passing Test

The assessments will have the same order for every participant regardless of the interventional arm allocation and will be conducted as follows: (1) LSPT numbers 1; (2) LSST numbers 1; (3) LIST-P; (4) LSPT numbers 2; and (5) LSST number 2 (Fig. 4).

**Fig. 5:**
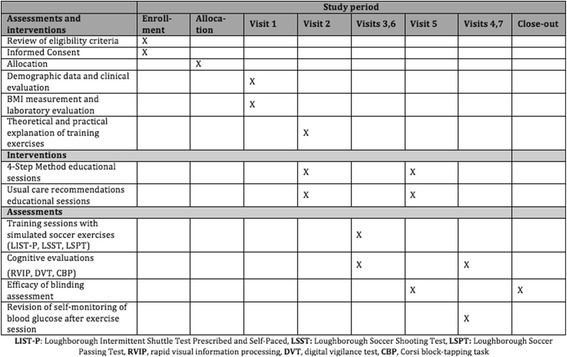
SPIRIT flow diagram. (A summary of the planned study interventions and assessments)

Additionally, as exploratory outcomes we will also assess cognitive functions, given that hypoglycemia has long been thought to influence these functions in people with diabetes mellitus. A field study of school-aged children with T1D has shown that low blood glucose level impairs patients’ sports performance and produces detrimental cognitive effects, as measured by math test performance [8]. In this sense we set forth to evaluate, as a secondary outcome, the effect of the 4-step method on the cognitive performance of the subjects, related also to the frequency of hypoglycemic events and to the performance in soccer indexed by the tests described. We hypothesize that besides showing an adequate safety profile (same or reduced number of hypoglycemic events), the 4-step method will also allow the subjects to achieve a better performance both in soccer and in cognitive testing (see Fig. 5 for a summary of the planned study interventions and assessments).

1. Stroop test
  The Stroop test is a commonly used neuropsychological tool that measures selective attention and mental processing speed among the cognitive functions that could be affected by attentional fatigue [22]. Campbell et al. utilized the Stroop test in a clinical trial that evaluated participants with T1D during physical activity in a sports camp. The investigators found that reading and color recognition ability were susceptible to impairment as a result of hypoglycemia [12].
  Commercially available Stroop Color and Word Tests (WPS Publish, Alaska, USA) will be used to assess cognitive processing in our participants. Scores will be determined for reading ability, color recognition, and interference; the latter will be used as an index of the subject’s capacity to maintain attention during the interference caused by the color changes in the typed words [23].
  Stroop testing will take place immediately following each soccer training session and during the morning evaluation at 8:00 a.m. on the next day. Each of these tests will take 2 to 5 min (time limit of 45 s per slide) for the participants to complete. Participants will be asked to complete the test using three slides: (1) read as many colored words as possible in 45 s; (2) name as many colors of a group of “X” letters as possible in 45 seconds; and (3) name the color in which a word is printed (e.g., “blue”, “green”, or “red” ink) and not the word itself, which is a name of a color (e.g., the word “red” printed in blue ink instead of red ink). Naming the color of the word during this “interference” takes longer and is more prone to errors than when the color of the ink matches the word that names the color [16]. Reading, color naming, and interference scores will be converted to *T* scores according to the manual of Golden and Stroop for further analyses.
2. Digit vigilance test
  The digit vigilance test (DVT) works as a measure of sustained attention and psychomotor speed. Participants will be asked to cross out a specific target number (6) as quickly as possible, which appears randomly within 59 rows of 35 single digits on two pages. The examiner will observe the subject's performance on the demonstration rows and then on the first five rows of the test to determine whether corrective feedback is required to discourage an inefficient or poorly motivated response style (e.g., going too fast and missing numbers or going too slow and focusing attention on each individual number). Total time and errors of omission will be recorded. For the first (practice) administration, all participants will be asked to complete the standard format (target = 6) [24].
3. Corsi block-tapping task
  The Corsi block-tapping task (CBT) is widely used for the assessment of visual-spatial short-term memory, both in clinical practice and in experimental research settings. Basically, it is a span task and, as such, a visual-spatial analog to the digit span, as an index of verbal short-term memory. The examiner taps a sequence of blocks, which the participant must repeat subsequently in the correct sequential order. By increasing the length of the sequences, the capacity of the visual-spatial short-term memory can be measured [25].
  The participant will be seated in front of the examiner, who subsequently will tap the cubes starting with a sequence of two blocks. Two trials will be given per block sequence of the same length. If at least one of these is repeated correctly, the next two trials of a sequence of an increased length will be administered. The cubes will be touched with the index finger at a rate of approximately one cube per second (with no pauses between the individual cubes). The participant will have to tap the cube sequences in the same order immediately after the examiner finishes. The sequences will gradually increase in length. The test will be terminated if the participant fails to reproduce two sequences of equal length. Only a completely, correctly repeated sequence will be scored as correct; self-corrections will be permitted.
4. Rapid visual information processing task
  The rapid visual information processing task (RVIP) is a serial detection task used to probe visual sustained attention and working memory processes. The design of the task consists of a stream of single digits (Arabic numerals 1–9) presented at a rate of 100–110 per minute. Participants are required to detect targets comprising a sequence of three consecutive odd or three consecutive even numbers. In a typical paradigm, targets are presented at a rate of four per 30 s. The critical performance variables include the number of correct responses (“hits”), the reaction time for hits, and the number of errors of commission (response in the absence of a target) [26].
  This RVIP version will be programmed using e-prime (Version 1.1. Psychology Software Tools, Inc., www.pstnet.com). White numbers between 0 and 9 (excluding 1) will appear in the center of a black computer screen. Each number is presented for 600 ms without a time gap between two succeeding numbers. The participant will have to detect three target sequences (“2–4–6”, “3–5–7”, and “4–6–8”) and respond to them (within 1800 ms after the onset of the last number) by pressing the space bar on the keyboard when the last number of a target sequence appears. The three target sequences will be displayed on the screen during the whole session in order to help the participants remember.
  Each block will be composed of 150 numbers that appear in a pseudo-random and fixed order; four target sequences will be included in 50 numbers. There will be 72 target sequences that appear during the 9-min test session. In order to prevent a sequence effect, six different RVIP versions will be created by varying these six blocks and then balancing them and pseudo-randomly assigning them to the participants. Hits (H), false alarms (FA) and reaction time (RT) will be recorded by the computer. All RTs shorter than or equal to 100 ms will be excluded from further analyses.

In a second step, the sensitivity A, measuring a subject's ability to distinguish targets and non-targets:

$$ \left(\mathrm{A} = 0.5 + \left[\mathrm{P}\left(\mathrm{H}\right) - \Big(\mathrm{P}\left(\mathrm{FA}\right) + \left(\left(\mathrm{P}\left(\mathrm{H}\right) - \mathrm{P}\left(\mathrm{FA}\right)\right)2\right]/\right[4\mathrm{P}\left(\mathrm{H}\right)\left(1 - \mathrm{P}\left(\mathrm{FA}\right)\right)\right), $$

the response criterion B, reflecting the individual's response tendency:

$$ \Big(\mathrm{B} = \left[\mathrm{P}\left(\mathrm{H}\right)\left(1 - \mathrm{P}\left(\mathrm{H}\right)\right) - \mathrm{P}\left(\mathrm{FA}\right)\left(1 - \mathrm{P}\left(\mathrm{FA}\right)\right)\right]/\Big[\mathrm{P}\left(\mathrm{H}\right)\left(1 - \mathrm{P}\left(\mathrm{H}\right)\right) + \mathrm{P}\left(\mathrm{FA}\right)\left(1 - \mathrm{P}\left(\mathrm{FA}\right)\right), $$

and the SD for the mean RT of each individual will be calculated. Sensitivity A, the number of hits, the SD and the mean RT will be evaluated from two perspectives: the level (e.g., the mean value of A over 9 min) and the time course (e.g., the mean value of A for the first 3 min, the second 3 min and the last 3 min). The number of FA and the response criterion B will be only evaluated from the perspective of level. The SD and the mean RT will only be computed for the hits [27].

### Sample size

We estimated a sample size of 24 participants per group, yielding a total sample size of 48 participants. However, because of the crossover design of this trial we will only need a total of 24 participants, as each of them will participate in both interventional arms. We used the following parameters for the sample size calculation: an alpha value of 0.05, power of 80%, two different intervention groups with a crossover design, and a minimal clinically important difference between the groups compared of 40% in the frequency of hypoglycemic events. Considering also a 15% dropout rate during the clinical trial, we obtained a required sample size of 28 participants for each group.

### Randomization

We will use block-stratified randomization, with random 2 − 4 sized blocks and stratified by gender. A statistician not related to the protocol will generate the randomization list using a web-based method (http://www.randomizer.org) that will randomize the order of the interventions assigned. We will then have sequentially numbered, sealed, opaque envelopes with the allocations inside, in order to maintain allocation concealment throughout the randomization process. After the patient is consented and enrolled in the trial, an un-blinded endocrinologist (also in charge of the educational sessions) will open the assigned envelope, allocate the subject to the group presented and coordinate with the pharmacy and nutritional department for the preparation of the insulin and dietary modifications needed with respect to the interventional arm assigned.

As mentioned before, the main objective that guides this exercise protocol is not to demonstrate differences in the response of subjects with T1D to different exercise modalities, such as continuous versus intermittent exercise. On the contrary, we want to evaluate the impact, in terms of safety and performance, of a protocol that sets forth insulin dosage along with carbohydrate intake diet modifications, compared with usual-care recommendations stated by guidelines for standard of care. This being said, it is worth adding that other trials [2, 10, 11] have evaluated the response of subjects to the 4-step method, so we would be increasing knowledge of this particular intervention.

### Blinding

In this trial, both participants and assessors will be blinded. Likewise, the statistical team in charge of data analysis will not know the real allocation of the participants and will handle data as “intervention A” and “intervention B”. In order to assure adequate blinding of the participants, we developed a sham 4-step method control intervention whereby we will administer the usual ADA recommendations masked in a 4-step method scheme similar to the real intervention (see Table 2). In this way, participants will feel that both are experimental trials even though they will be receiving different insulin and dietary regimens. Furthermore, all the assessors in charge of applying the soccer and cognitive evaluations will also be blinded until the end of the trial. We will have an un-blinded group of physicians that will be in charge of assigning patients to their allocations, applying and explaining the therapeutic and dietary modifications for each intervention, and monitoring glucose parameters and dietary or physical activity related adverse effects during the trial. Finally, before switching groups and at the end of the trial, we will assess the efficacy of blinding using a questionnaire that will be administered to the participants.

### Data collection

We will assemble a folder for each participant containing the data collection sheet that will include information acquired at the first visit on demographic data, clinical history, basal laboratory results, and the simulated soccer tests and cognitive function evaluation scores.

Blood glucose levels will be documented with glucometers. Given the simultaneous participation of several participants with T1D in the training sessions, five of these devices will be available for the research team in case of an accident or malfunction of an individual system. Glucometers *Accu-Check Nano* will be calibrated using the Accu-Check Smartview Glucose Control Solution every time and immediately before delivering them to each subject. Using this system, we will be able to identify if the glucometer is giving a correct value within a range previously established for the protocol. In the given case that the result is not within the desired range, then the device will no longer be used in the trial At the end of the 24-h follow up, the investigation team will check the recording after the training sessions and compare it with the finger stick results.

### Statistical methods

In order to evaluate glucose control, we will also compare the two groups, using either Student’s paired *t* test or the Wilcoxon-Mann-Whitney test, in terms of CGM parameters that include: 24-h mean glucose levels, SD of 24-h glucose levels, area under the curve for 24-h glucose, mean amplitude of glycemic excursions, number of episodes of hypoglycemia (<60 mg · dL^-1^), time in hypoglycemia (<60 mg · dL^-1^) during a 24-h period, time in hyperglycemia (>180 mg · dL^-1^) during a 24-h period, and range of postprandial glucose increase. The range of glucose increase (from pre-meal to post-prandial peak levels) after breakfast, lunch, and dinner, will also be compared between the interventional groups, using either repeated measures analysis of variance (ANOVA) or the Kruskal-Wallis test, depending on the normality of the data distribution.

We will assess for data from the LIST-P, LSPT, and LSST, and the Stroop, DVT, DBT, and RVIP test scores obtained for each assessment session, for normality of distribution using histogram analysis, the Shapiro-Wilk test and the Kolmogorov-Smirnov test. We will use a logarithmic transformation for the correction of non-normal data and will subsequently test for the differences between groups using Student’s paired *t* test. In order to assess if the order of the intervention modified the performance of individual participants, we will calculate the difference of the scores obtained between the two types of interventions (real intervention minus sham 4-step method) and then we will define an intervention indicator variable that will take the value of 1 if the subject started with the real 4-step method, and 0 if the subject started with the sham 4-step method. We will then fit a linear regression model with the difference calculated as the response variable and the intervention indicator along with other relevant covariates (age, gender, BMI) as the independent variables. Moreover we will test for time effect over the performance of the participants in a time-varying regression model. Additionally, Pearson correlation coefficients will be calculated between each of the scores and the pre-exercise blood glucose level, searching for linear correlation between blood glucose values and the LIST-P, LSPT, LSST, and Stroop test scores.

In the event that the data do not follow the normal distribution even after the log-transformation, we will conduct non-parametric analyses using the Wilcoxon-Mann-Whitney test and calculate Spearman correlation coefficients for the comparison between the two groups of the performance scores in soccer and cognitive tests, and for the linear correlation analysis, respectively. A *p* value <0.05 will be considered significant for the comparison of the scores.

Additionally, we will conduct exploratory analyses by modeling the performance scores in soccer evaluations adjusted by age, years since T1D diagnosis, HbA1c level, weekly physical activity hours and Stroop test scores using linear multiple regression. A *p* value <0.10 will be considered significant to introduce a variable into the multivariate regression model. The number of hypoglycemic events and the percentage of other adverse effects listed previously will also be compared among groups using Student’s *t* test and the Fisher exact test, respectively. All of the statistical analysis will be done using Stata software (StataCorp LP® 13.1).

### Ethical considerations

The principal investigator and the research assistant for the trial will be responsible for enrolling and contacting participants and conducting the informed consent process. All participants will be assigned an individual code and any personal health information (PHI) that may identify the participants will be protected, and only the research team will have access to it. Any relevant changes in the study protocol and/or the informed consent will be submitted to the independent review board either as protocol deviation report or as protocol amendments. The protocol will be registered in www.isrctn.com before starting with the trial.

### Consent and permissions

Participants that fulfill the eligibility criteria will be defined as potential participants of the clinical trial, and will receive the informed consent through either email or physical mail. Afterwards, an in-person meeting will be scheduled and during this session, the principal investigator will explain the methodology and the design of the study in detail. The subjects will also be asked to give written informed consent during this visit, after asking questions and/or expressing their concerns.

### Adverse event monitoring

An un-blinded group of physicians and physical trainers will be in charge of monitoring glucose derangements and physical injuries that may arise during the assessments. We have prepared protocol-based treatment and dietary interventions that follow guidelines established for both the usual recommendations and the 4-step method groups, which will be executed in the case of hyperglycemic or hypoglycemic events. Also, we will record any adverse events observed during the execution of the trial and, if required, report them directly to our Ethics Committee for further evaluation. Additionally, our technical team will check and calibrate each of the glucometers and CGM devices before using them in any of the protocol assessments.

## Discussion

During clinical trials the 4-step method has been demonstrated to optimize blood glucose levels during and after exercise by making mealtime adjustments to both rapid-acting and basal insulin and to pre-exercise and post-exercise carbohydrate composition [2]. The first step of this method protects individuals with T1D from concomitant and early exercise-induced hypoglycemia [6], the second step prevents a rapid rise in blood glucose because of an overcorrection with food using a low-GI carbohydrate meal [7], and the third and fourth steps are intended to guard against late-onset exercise-induced hypoglycemia after physical activity [2, 11].

Intermittent exercise, such as soccer, is more demanding on the body than continuous exercise of the same average intensity [21]. The 4-step method has never been tested in a clinical trial setting for the evaluation of intermittent forms of exercise, such as soccer.

We expect that this protocol will stimulate the development and execution of clinical trials to evaluate dietary and pharmacological interventions that will help patients with T1D engage in physical activity, reduce the risk of glycemic derangements while conducting exercise, and at the same time obtain the best physical and cognitive performance possible.

### Trial status

1. Date of first enrollment: 1/8/2017
2. Target sample size: 28 patients
3. Recruitment status: pending

## Additional file

Additional file 1: SPIRIT 2013 checklist. (DOC 122 kb)

## Abbreviations

ADA: American Diabetes Association
ANOVA: Analysis of variance
BMI: Body mass index
CBP: Corsi block-tapping task
CGM: Continuous glucose monitor
CHO: Carbohydrates
DPP-4: Dipeptidyl peptidase 4
DVT: Digital vigilance test
FAS: Felt arousal scale
FS: Feeling scale
GI: Glycemic index
GLP1: Glucahon like peptide 1
HbA1c: Glycated hemoglobin
HR: Heart rate
IRB: Institutional Review Board
LIST-P: Loughborough Intermittent Shuttle Test Prescribed and Self-Paced
LSPT: Loughborough Soccer Passing Test
LSPT: Loughborough Soccer Passing Test
LSST: Loughborough Soccer Shooting Test
RPE: Rating perceived exertion scale
RVIP: Rapid visual information processing
SD: Standard deviation
SGLT-2: Sodium glucose cotransporter 2
T1D: Type 1 diabetes mellitus

## References

[1] Robertson K, Riddell MC, Guinhouya BC, Adolfsson P, Hanas R (2014). Exercise in children and adolescents with diabetes. Pediatr Diabetes..

[2] Campbell M, Walker M, Bracken R, Turner D, Stevenson E, Gonzalez J, Shaw J, West D (2015). Insulin therapy and dietary adjustments to normalize glycemia and prevent nocturnal hypoglycemia after evening exercise in type 1 diabetes: a randomized controlled trial. BMJ Open Diabetes Res Care..

[3] McMahon SK, Ferreira LD, Ratnam N (2007). Glucose requirements to maintain euglycemia after moderate-intensity afternoon exercise in adolescents with type 1 diabetes are increased in a biphasic manner. J Clin Endocrinol Metab..

[4] Tschakert G, Hofmann P (2013). High-intensity intermittent exercise: methodological and physiological aspects. Int J Sports Physiol Perform.

[5] Moser O, Tschakert G, Mueller A, Groeschl W, Pieber TR, Obermayer-Pietsch B, Koehler G, Hofmann P (2015). Effects of high-intensity interval exercise versus moderate continuous exercise on glucose homeostasis and hormone response in patients with type 1 diabetes mellitus using novel ultra-long-acting insulin. PLoS One.

[6] Nevill M, Garrett A, Maxwell N, Parsons K, Nowitz A (1995). Thermal strain of intermittent and continuous exercise at 10 °C and 35 °C. J Physiol..

[7] Bangsbo J (1994). The physiology of soccer - with special reference to intense intermittent exercise. Acta Physiol Scand Suppl..

[8] Shi X, Gisolfi C (1998). Fluid and carbohydrate replacement during intermittent exercise. Sports Med..

[9] Hargreaves M, Dillo P, Angus D, Febbraio M (1996). Effect of fluid ingestion on muscle metabolism during prolonged exercise. J Appl Physiol.

[10] Bally L, Zueger T, Buehler T, Dokumaci AS, Speck C, Pasi N, Ciller C, Paganini D, Feller K, Loher H, Rosset R, Wilhelm M, Tappy L, Boesch C, Stettler C (2016). Metabolic and hormonal response to intermittent high-intensity and continuous moderate intensity exercise in individuals with type 1 diabetes: a randomised crossover study. Diabetologia.

[11] Yardley JE, Colberg SR (2017). Update on management of type 1 diabetes and type 2 diabetes in athletes. Curr Sports Med Rep.

[12] Kelly D, Hamilton J, Riddell M. Blood glucose levels and performance in a sports camp for adolescents with type 1 diabetes mellitus: a field study. Int J Pediatr. 2010. doi:10.1155/2010/21616. 10.1155/2010/216167 PMC2929497 20811595

[13] West D, Morton R, Bain S, Stephens J, Bracken R (2010). Blood glucose responses to reductions in pre-exercise rapid-acting insulin for 24 h after running in individuals with type 1 diabetes. J Sports Sci.

[14] Campbell M, Walker M, Trenell M, Jakovljevic D, Stevenson E, Bracken R (2013). Large pre- and post exercise rapid-acting insulin reductions preserve glycemia and prevent early- but not late-onset hypoglycemia in patients with type 1 diabetes. Diabetes Care..

[15] Campbell M, Walker M, Trenell M, Stevenson E, Turner D (2014). A low–glycemic index meal and bedtime snack prevents postprandial hyperglycemia and associated rises in inflammatory markers, providing protection from early but not late nocturnal hypoglycemia following evening exercise in type 1 diabetes. Diabetes Care..

[16] Colberg S, Sigal R, Yardley J, Riddel M, Dunstan D, Dempsey P (2016). Physical activity/exercise and diabetes: a position statement of the American Diabetes Association. Diabetes Care..

[17] Ali A, Foskett A, Gant N (2014). Measuring intermittent exercise performance using shuttle running. J Sports Sci.

[18] Borg G (1973). Perceived exertion: A note on “history” and methods. Med Sci Sports Exerc..

[19] Astorino T, Cottrell T, Talhami Lozano A (2012). Effect of caffeine on RPE and perceptions of pain, arousal, and pleasure/displeasure during a cycling time trial in endurance trained and active men. Physiol Behav.

[20] Ekkekakis P, Parfitt G, Petruzzello SJ (2011). The pleasure and displeasure people feel when they exercise at different intensities: decennial update and progress towards a tripartite rationale for exercise intensity prescription. Sports Med.

[21] Ali A, Williams C, Hulse M, Strudwick A, Reddin J, Howarth L (2007). Reliability and validity of two tests of soccer skill. J Sports Sci.

[22] Stroop J (1935). Studies of interference in serial verbal reactions. J Exp Psychol..

[23] Golden, Charles J., Shawana M. Freshwater, and Golden Zarabeth. Stroop color and word test children's version for ages 5-14: a manual for clinical and experimental uses. Wood Dale, Ill: Stoelting. 2003.

[24] Kelland D, Lewis R (1996). The Digit Vigilance Test: reliability, validity, and sensitivity to diazepam. Arch Clin Neuropsychol.

[25] Kessels R, van Zandvoort M, Postma A, Kappelle J, de Haan E (2000). The Corsi block-tapping task: standardization and normative data. Appl Neuropsychol.

[26] Neale C, Johnston P, Hughes M, Scholey A (2015). Functional activation during the rapid visual information processing task in a middle aged cohort: an fMRI study. PLoS One.

[27] Hilti C, Hilti L, Heinemann D, Robbins T, Seifritz E, Cattapan-Ludewig K (2010). Impaired performance on the rapid visual information processing task (RVIP) could be an endophenotype of schizophrenia. Psychiatry Res.

